# From Oilseed Waste to High-Value Bioactives: Deep Eutectic Solvents as Sustainable Refining Media

**DOI:** 10.3390/ijms27167125

**Published:** 2026-08-08

**Authors:** Marcelina Mazur, Kristina Radošević, Marina Cvjetko Bubalo, Višnja Gaurina Srček, Ivana Radojčić Redovniković

**Affiliations:** 1 Department of Food Chemistry and Biocatalysis, Wrocław University of Environmental and Life Sciences, 25 Norwida St., 50-375 Wrocław, Poland; 2 Laboratory for Cell Technology, Application and Biotransformation, University of Zagreb Faculty of Food Technology and Biotechnology, Pierottijeva 6, 10000 Zagreb, Croatia; kristina.radosevic@pbf.unizg.hr (K.R.); marina.cvjetko.bubalo@pbf.unizg.hr (M.C.B.); visnja.gaurina.srcek@pbf.unizg.hr (V.G.S.); ivana.radojcic.redovnikovic@pbf.unizg.hr (I.R.R.)

**Keywords:** oilseed by-products, DES extracts, phenolics, proteins, tocopherols, carbohydrates

## Abstract

The global oil-processing industry generates substantial quantities of by-products and secondary streams, including oilseed cakes, pomaces, hulls, and wastewaters, which remain largely underutilized despite being rich sources of high-value bioactive compounds. The development of sustainable strategies for the valorization of these residues is increasingly recognized as a key component of circular bioeconomy and biorefinery frameworks. In this context, deep eutectic solvents (DESs) have attracted considerable attention as a new generation of designer solvents owing to their tunable physicochemical properties, low vapor pressure, ease of synthesis, and potential environmental compatibility. This review critically discusses the current state of knowledge regarding the application of DESs in the processing and valorization of oil industry by-products. Particular emphasis is placed on the relationship between DES composition, physicochemical characteristics, and extraction performance. Recent advances in the recovery of phenolic compounds, proteins, saccharides, and tocopherols from oilseed-derived residues are comprehensively examined, including the integration of DESs with intensified extraction techniques such as microwave-, ultrasound-, and ohmic-assisted extraction. Furthermore, the role of DESs in oil purification processes and the treatment of technological waste stream is evaluated. Emerging evidence indicates that DES-based systems not only enhance extraction efficiency and selectivity but may also improve the stability, bioaccessibility, and purity of the recovered compounds. Finally, the opportunities and challenges associated with the implementation of DES-based technologies within integrated biorefinery schemes are discussed, including solvent recovery, product scalability, sensory acceptability, and regulatory considerations. The available literature demonstrates that DESs constitute a versatile platform for the sustainable valorization of oil-processing residues, supporting the transition from conventional waste management approaches toward resource-efficient and circular production systems.

## 1. Introduction

**Figure 1:**
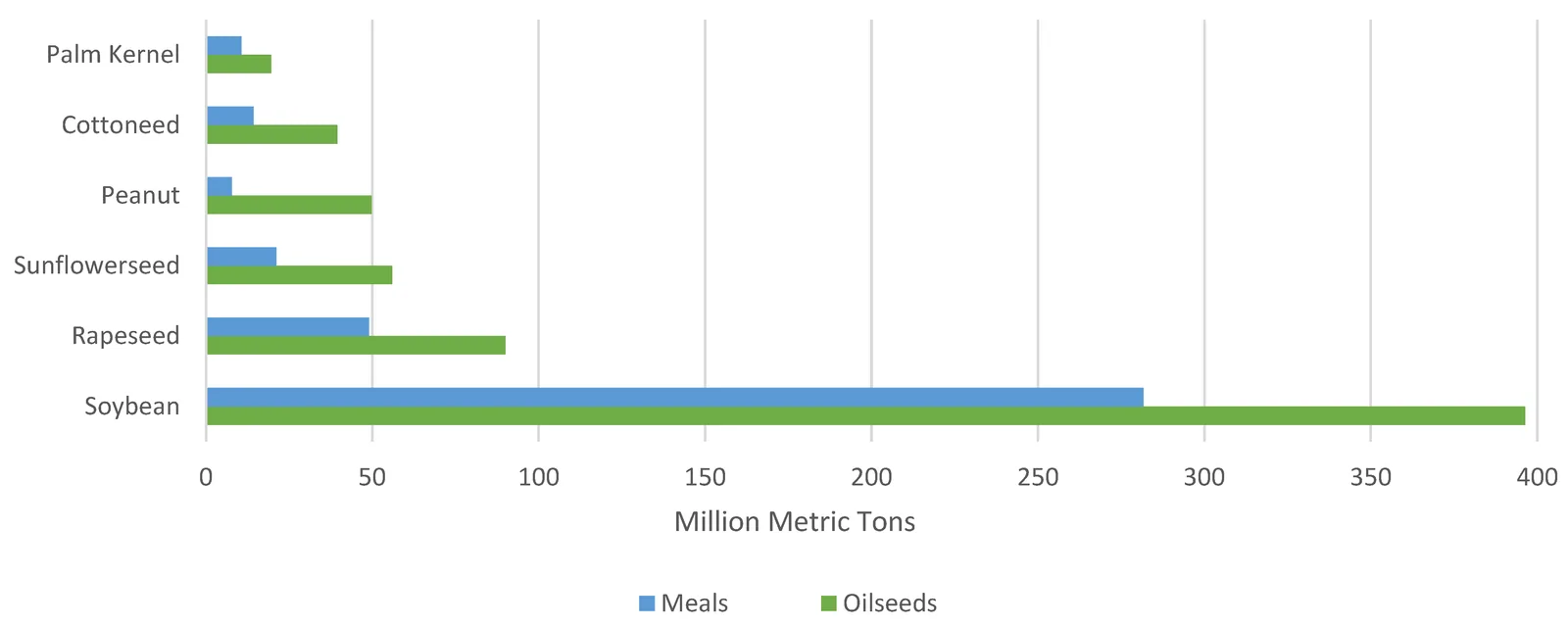
Oilseeds and meal world production in 2024/25 (Million Metric Tons) [3].

Global production of vegetable oils has increased significantly over recent decades due to the growing demand from the food, feed, biofuel, cosmetic, and pharmaceutical industries. Oilseed crops such as rapeseed, soybean, sunflower, palm kernel, flaxseed, and camelina constitute some of the most important agricultural commodities worldwide [1]. According to recent agricultural statistics, soybean and palm oil dominate the global vegetable oil market, while rapeseed and sunflower oils are particularly important in Europe [2]. The continuous expansion of the oilseed processing sector has led not only to increased oil production but also to the generation of substantial amounts of by-products and processing residues (Figure 1).

The processing of oil-bearing crops involves several technological steps, including seed cleaning, conditioning, dehulling, mechanical pressing, solvent extraction of residual oil and oil refining [4,5,6]. Depending on the extraction method, different by-products are generated, such as oilseed meals, press cakes, hulls, gums, soapstocks, and deodorization distillates. Traditionally, many of these residues have been used as low-value animal feed [7,8] or disposed of as industrial waste [9]. However, increasing attention has recently been paid to their chemical composition and potential as renewable sources of valuable bioactive compounds [10,11].

The composition of oil industry by-products varies depending on the botanical origin of the raw material and the processing technology employed (Table 1). Nevertheless, these residues generally contain substantial amounts of proteins, dietary fiber, residual oils, and a wide range of bioactive constituents, including polyphenols, tocopherols, phytosterols, and phenolic acids. Owing to this rich chemical profile, oilseed by-products exhibit significant antioxidant, anti-inflammatory, and other health-promoting properties, making them promising raw materials for various applications in the food, nutraceutical, pharmaceutical, and cosmetic sectors [12,13,14].

In response to the growing demand for sustainable and environmentally friendly technologies, green extraction approaches have gained considerable interest. In particular, Deep Eutectic Solvents (DESs) have emerged as promising alternatives to traditional solvents due to their low toxicity, biodegradability, tunable physicochemical properties, and high extraction efficiency. These systems offer new opportunities for the sustainable valorization of oilseed processing by-products within the framework of circular bioeconomy and green chemistry principles. Accordingly, this review focuses on recent advances in the valorization of oilseed processing by-products using DESs. The literature included in this review was selected based on its relevance to oilseed by-products, green extraction technologies, the recovery of bioactive compounds, and sustainable processing strategies. Particular emphasis was placed on recent peer-reviewed publications reflecting the latest developments in the field, while relevant studies on DES chemistry and extraction mechanisms were included to provide the necessary scientific background. To place these developments in an industrial context, current data on global oilseed production were complemented with reports from international organizations.

## 2. Characteristics of Deep Eutectic Solvents in the Context of Extraction of Bioactive Compounds from Oilseed Processing By-Products

Although DESs were initially described as mixtures of quaternary ammonium salts (hydrogen bond acceptor—HBA) and hydrogen bond donors—HBDs [20], a more general definition classifies them as mixtures of Brønsted and Lewis acids and bases capable of forming extensive hydrogen-bond networks. These interactions cause a significant deviation from ideal mixture behavior, resulting in a substantial depression of the freezing point compared to the individual components [21]. The concept has therefore been broadened to include a wide range of acid–base systems.

For the extraction of bioactive compounds from plant materials, natural deep eutectic solvents (NADESs) are most commonly employed. These solvents are prepared exclusively from naturally occurring metabolites, including organic acids, amino acids, sugars, and polyols. NADESs have attracted considerable attention as extraction media due to their low toxicity, high biocompatibility, biodegradability, and ability to efficiently solubilize a wide range of phytochemicals.

The physicochemical properties of DESs can be tailored by selecting appropriate HBA/HBD combinations and adjusting their molar ratios, making them highly versatile extraction media for recovering bioactive compounds from complex plant matrices such as oilseed processing by-products. Additionally, most DESs retain the bioactivity of target compounds, which further enhances their potential for applications in the food, pharmaceutical, and cosmetic industries [22]. The extraction performance of DESs strongly depends on both their composition and the characteristics of the plant matrix. Choline chloride-based NADESs are among the most widely studied systems and have shown high efficiency in the extraction of polar compounds from plant materials [23,24], while hydrophobic DESs (HDES) are more attractive for liquid–liquid extraction of non-polar organic and inorganic molecules from aqueous environments [25]. The selectivity of DESs is primarily governed by the chemical compatibility between the solvent and the target molecules according to the “like dissolves like” principle. By carefully selecting the HBA, HBD, and their molar ratio, the polarity, hydrogen-bonding capacity, viscosity, and acidity of the solvent can be precisely tailored to maximize affinity toward specific classes of bioactive compounds. For instance, DESs based on organic acids, sugars, or polyols exhibit high extraction efficiency for phenolic acids and flavonoids because their abundant hydroxyl and carboxyl groups form strong hydrogen-bonding interactions with phenolic functional groups [26,27,28]. Conversely, less polar or hydrophobic DES formulations display greater affinity for more lipophilic constituents, such as carotenoids, tocopherols, and other less polar phytochemicals. This tunability of solvent composition enables the rational design of DESs for the selective recovery of target bioactive compounds from complex oil-processing by-product matrices [29,30].

Proteins are considerably more sensitive to extraction conditions than phenolic compounds with respect to preserving their biological activity. Therefore, the optimal DES should not only provide high extraction efficiency but also maintain protein functionality, minimize denaturation, and facilitate downstream recovery. For this reason, many studies focus on the entire extraction–purification process rather than on solvent power alone [31].

### 2.1. Physicochemical Properties of DESs

The effectiveness of deep eutectic solvents in the valorization of vegetable oil industry by-products is strongly influenced by their physicochemical properties. Given the heterogeneous composition of oilseed cakes, meals, pomace, and olive processing residues, the extraction performance of DES systems depends on a complex interplay between solvent polarity, viscosity, water content, acidity, and thermal stability. Table 2 presents the density and viscosity of representative DESs, highlighting the variability of these properties depending on solvent composition.

Solvent polarity is a key factor governing extraction selectivity. Hydrophilic DESs composed of choline chloride (ChCl), betaine, amino acids, sugars, polyols, or organic acids exhibit high affinity toward polar and moderately polar phytochemicals, including phenolic acids, flavonoids, glucosinolates, and phytates. Conversely, hydrophobic DESs are more suitable for the recovery of lipophilic constituents, such as residual triglycerides, tocopherols, phytosterols, carotenoids, and other lipid-soluble antioxidants [40,41]. The extraction efficiency of DESs is strongly influenced by the hydrogen-bonding interactions between the solvent and the target compound. Studies comparing different DES systems, including Ethaline (ChCl:Ethylene glycol) and Glyceline (ChCl:Glycerol), Oxaline (ChCl:Oxalic acid), and Reline (ChCl:Urea), demonstrated that solute partitioning into DESs increases when solvation is thermodynamically favorable and strong hydrogen bonds can be formed with the DES components [42]. When the tested compounds were butanoic acid, 1-pentanol, 2-pentanone, ethyl acetate, phenol, benzyl alcohol, and cyclohexanol, the type of DES was found to have a greater impact on the extraction of less polar compounds, whereas the extraction of more polar solutes was less dependent on DES composition. Furthermore, a correlation between extraction efficiency and the pKa of the solute highlighted the key role of hydrogen-bonding interactions in the extraction process. Similar trends were observed during the extraction of phenolic compounds from a lipid matrix, where compounds with lower pKa values, such as phenolic acids, exhibited higher extraction efficiencies than less acidic molecules [42,43,44,45,46].

The relatively high viscosity of many DES systems remains one of their principal limitations. Elevated viscosity can impair solvent penetration into plant tissues and reduce mass-transfer rates, particularly in lignocellulose-rich matrices characterized by compact cell-wall structures. This limitation is especially relevant for oilseed processing residues, which frequently contain substantial amounts of dietary fiber, hull fractions, and protein–phenolic complexes [47]. To overcome these mass-transfer limitations, controlled amounts of water are commonly added to DESs to reduce viscosity, promote matrix swelling, and enhance diffusion kinetics [48,49]. In practice, the presence of water is almost unavoidable, as most DESs are highly hygroscopic and difficult to dry completely [50]. Furthermore, maintaining strictly anhydrous conditions is challenging even at laboratory scale and would become a significant technical and economic constraint in industrial extraction processes. As a result, DES-based extractions are typically performed in the presence of a defined amount of water. However, water content must be carefully optimized. While moderate water addition improves extraction performance, excessive dilution can disrupt the hydrogen-bonding network responsible for eutectic behavior, thereby reducing the unique solvation properties and extraction efficiency of the solvent system. Therefore, achieving an appropriate balance between viscosity reduction and preservation of DES structure is essential for efficient extraction [51,52].

The apparent acidity or basicity of DES formulations also significantly affects extraction outcomes. Acidic systems based on organic acids may facilitate the release of bound phenolic compounds and promote partial disruption of plant cell-wall structures, thereby enhancing extraction yields. However, highly acidic media may negatively affect protein integrity and the stability of sensitive bioactive compounds. In contrast, neutral or mildly acidic systems are generally considered more appropriate for food-oriented applications and protein recovery processes [40]. The study of Sombutsuwan et al. demonstrated that the acidity or alkalinity of DESs strongly influences the extraction of bioactive compounds from defatted rice bran [53]. Acidic DES based on ChCl:Lactic acid (pH 0.42) were particularly effective for the recovery of phytic acid, reducing sugars, and phenolic acids, whereas the alkaline DES based on K_2_CO_3_:Glycerol (pH 11.21) yielded the highest concentrations of both phenolic acid and proteins. The authors observed that DESs characterized by less extreme pH values resulted in lower extraction efficiencies, likely due to a less effective disruption of plant cell walls, which consequently limited the release of phenolic compounds and other bioactive constituents [53]. A comparative analysis of DESs revealed that the chemical nature of the HBD plays a key role in determining both extraction efficiency and selectivity toward specific phenolic compounds. In alcohol-based DESs, short carbon chains and a high degree of hydroxylation generally favored phenolic extraction. For organic acid-based DESs, extraction efficiency depended largely on the degree of carboxylation and the structural form of the target compound. Highly carboxylated DESs were more effective for the extraction of aglycones, while less carboxylated systems favored glycosylated phenolics [28].

Although DESs are generally characterized by low volatility and favorable thermal stability, extraction temperature remains a critical parameter. Elevated temperatures can improve extraction kinetics and mass transfer but may simultaneously accelerate lipid oxidation, phenolic degradation, protein denaturation, and non-enzymatic browning reactions [54,55]. On the other hand, the behavior of DESs at elevated temperatures is also an important aspect to consider. Unlike ionic liquids, DESs undergo a progressive decomposition process initiated by the weakening of hydrogen-bond interactions, followed by the volatilization or degradation of their individual HBD and HBA components. Hydrogen bonding enhances the thermal stability of DESs and contributes to higher onset decomposition temperatures (T_onset_), while the thermal stability of the HBD is a key factor determining the overall stability of the system [56]. Understanding these thermal degradation mechanisms is essential for the rational design of DESs for practical applications. An important aspect of DES applicability is their thermal stability under operating conditions. Although many choline chloride-based DESs, such as Reline and Glyceline, exhibit T_onset_ above 160–180 °C in dynamic thermogravimetric analysis (TGA), these values do not necessarily reflect their long-term thermal stability. Several studies have shown that chemical transformations, including discoloration, changes in acidity, and partial decomposition of DES constituents, may occur at considerably lower temperatures (approximately 80–140 °C), depending on the DES composition and operating conditions. Consequently, the maximum continuous use temperature is often significantly lower than the T_onset_ value determined by TGA. For this reason, biomass pretreatment and extraction processes employing DESs are commonly conducted below 120 °C to minimize solvent degradation and preserve process efficiency [56,57,58]. These observations highlight the importance of complementing dynamic TGA measurements with long-term isothermal stability studies when assessing the practical thermal limits of DESs.

Overall, the extraction efficiency of DESs is governed by the complex interplay between their physicochemical properties and the molecular interactions established with target bioactive compounds. As discussed above, parameters such as polarity, viscosity, acidity/basicity, temperature, and water content determine the solvent environment and directly influence solute solubility, diffusion, and mass-transfer kinetics. Efficient extraction therefore requires a careful balance between solvent–solute affinity and transport phenomena. Strong hydrogen-bonding networks generally enhance the solubility and selectivity of target compounds; however, they also increase solvent viscosity, thereby restricting molecular diffusion and reducing mass-transfer rates during solid–liquid extraction. To overcome this limitation, water is commonly incorporated into DESs to partially disrupt the hydrogen-bonding network, decrease viscosity, and improve solvent penetration into plant matrices. At appropriate concentrations, this enhances mass transfer, facilitates the disruption of plant cell walls, promotes the release of intracellular constituents, and improves the dissolution of bioactive compounds into the solvent phase while preserving sufficient solvent–solute interactions [24,26,27,59]. Excessive water addition, however, further weakens the hydrogen-bonding network, resulting in reduced solvent–solute affinity, lower extraction selectivity, and diminished extraction efficiency. Consequently, the performance of DESs cannot be attributed to any single physicochemical parameter but rather to the synergistic effects of solvent polarity, hydrogen-bonding capacity, viscosity, acidity/basicity, water content, and operating conditions. Rational optimization of the HBA/HBD combination, molar ratio, water content, and extraction temperature is therefore essential to achieve an optimal balance between molecular interactions and mass-transfer efficiency, ultimately maximizing the selective recovery of bioactive compounds from oil-processing by-products.

### 2.2. Toxicity, Biodegradability and Environmental Aspects

Deep eutectic solvents are generally regarded as green solvents characterized by low toxicity and good biodegradability. These assumptions are mainly based on studies performed on DESs composed of choline chloride and various hydrogen bond donors. Numerous cytotoxicity studies conducted on mammalian cells, algae, and plants have shown that most DESs exhibit low-to-moderate toxicity, making them promising alternatives to conventional organic solvents and ionic liquids [60,61,62,63,64,65].

Radošević et al. demonstrated that the tested DESs exhibited lower toxicity than the investigated ionic liquids (ILs). The tested solvents also showed relatively low toxicity toward wheat in germination assays. The highest toxicity was associated with DESs containing oxalic acid as the HBD component, which the authors attributed mainly to the low pH of the system [60]. These observations indicate that the toxicity of DESs strongly depends on the nature of the hydrogen bond donor and the physicochemical properties of the resulting solvent.

Similarly, Paulo de Morais et al. investigated several DESs based on choline chloride and organic acids (acetic, citric, lactic and glycolic acids) using the Microtox® toxicity test on *Vibrio fischeri*. The authors reported that the studied DESs exhibited moderate toxicity, with the toxic effects being largely dependent on the acid concentration. Moreover, the tested DESs were found to be more toxic than the corresponding cholinium-based ionic liquids, including cholinium glycolate, cholinium acetate, cholinium dihydrogencitrate, and cholinium lactate [61].

Q. Wen et al. evaluated the toxicity of different DESs toward garlics and hydras and observed that all tested DESs and their individual components affected the growth and viability of both organisms. The toxic effects included shorter hydra lifetime, reduced root growth in garlic cloves, as well as deformation and disintegration of root tip cells. However, the authors also demonstrated that DESs were generally less toxic towards garlics and hydras than their individual components, suggesting that the extensive hydrogen-bonding network formed within the DES structure may reduce the bioavailability of toxic species. In addition, DESs based on choline chloride exhibited stronger inhibitory effects than those based on choline acetate, indicating that the type of cholinium salt plays an important role in determining toxicity [62].

Additional evidence of the low toxicity of DESs was provided by Inês João Ferreira et al., who investigated the effects of two DES systems, Betaine:Sorbitol:Water and Betaine:Glycerol, using zebrafish (*Danio rerio*) after intraperitoneal administration. The tested DESs and their individual components were evaluated at different concentrations. The results showed no significant toxicity up to concentrations of 5000 μM for Betaine:Sorbitol:Water and 3000 μM for Betaine:Glycerol, while the individual components were tested up to 1000 μM. Based on these findings, the authors concluded that both DES systems may be considered environmentally friendly solvents with low toxicological impact [63].

The biodegradability of DESs has also been extensively investigated. In most studies, biodegradation was evaluated using the Closed Bottle Test according to OECD guidelines [64]. This method measures the oxygen consumption of microorganisms in a closed aerobic system containing the tested compound and allows the determination of the degree of biodegradation over time. According to OECD criteria, a substance is considered readily biodegradable when at least 60% biodegradation is achieved within 28 days.

Radošević et al. demonstrated that all tested DESs exhibited biodegradation levels above 60% and therefore could be classified as readily biodegradable. The investigated systems showed the following biodegradability order: ChCl:Glycerol > ChCl:Glucose > ChCl:Oxalic acid. The highest biodegradation level reached 96%, whereas the lowest value was 68%. Furthermore, ChCl:Glycerol and ChCl:Glucose reached biodegradation levels of 84% and 66%, respectively, after only seven days, while ChCl:Oxalic acid exceeded 60% biodegradation after 14 days [60].

Similar observations were reported by Zampeti et al., who investigated DESs based on choline chloride and various organic acids, including oxalic acid, malonic acid, succinic acid, levulinic acid, citric acid, and malic acid. According to the Organisation for Economic Co-operation and Development (OECD) criteria, DESs composed of ChCl:Levulinic acid and ChCl:Malic acid could be classified as readily biodegradable because their biodegradability ratios exceeded 80% within five days. The highest and lowest biodegradation rates after five days were observed for ChCl:Levulinic acid (86.1%) and ChCl:Succinic acid (15.6%), respectively. Moreover, other tested DESs already exhibited biodegradation rates above 39.1% after five days, suggesting that they may also fulfill the OECD criteria for ready biodegradability after longer incubation periods. The authors also observed that structural factors significantly influenced DES biodegradability. For three out of the six tested DESs, increasing the alkyl chain length resulted in lower biodegradability. In contrast, the presence of hydroxyl groups within the DES structure appeared to enhance biodegradation, likely due to improved susceptibility to microbial degradation processes [65].

Overall, although DESs are frequently classified as environmentally friendly solvents, available studies indicate that their toxicity strongly depends on chemical composition, acidity, and the interactions between their components. Therefore, each DES system should be individually evaluated before its application.

The environmental aspects and sustainability of DESs depend not only on their toxicity and biodegradability, but also on their ability to be recovered, purified, and reused over multiple extraction cycles. These factors, together with extraction efficiency, also play a crucial role in determining the economic sustainability of DES-based extraction processes. Their large-scale implementation is strongly influenced by solvent regeneration efficiency, solvent loss during recovery, and the preservation of physicochemical properties during repeated use, which is discussed in more detail in Section 5. Briefly, recent studies indicate that many DESs can be recycled for multiple extraction cycles with relatively small losses in extraction performance, particularly when efficient purification strategies are applied [66,67]. Recovery efficiencies above 85–90% have been reported for several DES systems, while extraction efficiency is often maintained for 3–10 cycles depending on DES composition, purification method, and feed matrix [27,68]. Overall, the current evidence indicates that efficient recovery, purification, and water-content management can maintain extraction performance over multiple cycles and substantially improve the environmental profile of DES-based extraction systems.

Regarding the environmental profile of DES-based extracts, their safety assessment should be conducted not only for extracted biological active compounds and their functional properties but also for the potential presence of residual solvent traces in the final product. The final extract should be considered as a formulation containing both bioactive constituents and residual solvent components, since its biological activity may reflect the combined effects of both fractions. As already discussed, although DESs are generally regarded as safer alternatives to conventional volatile organic solvents (VOCs), their safety cannot be assumed solely from the toxicity profile of the individual constituents [69,70]. For that reason, residual solvent traces in final extract remain an important quality and safety consideration, especially from a pharmaceutical and nutraceutical perspective. Unlike VOCs, which are regulated under ICH Q3C guidelines [71], DESs are non-volatile and may persist in the final extract even after conventional drying procedures. Consequently, residual DES content should be quantified using appropriate analytical methods, such as HPLC, quantitative NMR, ion chromatography, or LC-MS, depending on the solvent composition. Also, residual DES content should be minimized and justified through toxicological evaluation rather than assuming negligible risk. Furthermore, the stability of the DES during the extraction process and storage of the final extract also should be considered. Recent studies have shown that certain choline chloride-based DESs may undergo slow decomposition even at room temperature [72], potentially generating degradation products with different toxicological properties. Yang et al. reported partial decomposition of ethaline into toxic chloromethane and dimethylaminoethanol at room temperature, limiting its sustainable advantage. This finding highlights the importance of assessing not only the initial DES composition, but also the chemical integrity of the solvent throughout processing and storage.

Overall, it can be concluded from the current literature that DES-derived extracts possess favorable safety profiles, particularly when NADES systems composed of food-grade components are employed. Nevertheless, regulatory acceptance of DES-extracted products will require comprehensive safety assessment, which should include solvent characterization before and after extraction, determination of residual solvent levels in the final product, and cytocompatibility testing of the complete extract formulation.

## 3. Deep Eutectic Solvents in the Extraction of Bioactive Compounds from Oilseed Processing By-Products

Oilseed processing by-products, including meals, cakes, and press residues, represent valuable sources of bioactive compounds such as phenolic acids, flavonoids, tocopherols, phytosterols, proteins and peptides. Conventional extraction of these compounds is commonly performed using organic solvents, including methanol, ethanol, acetone, or hexane [73,74,75,76]. However, increasing environmental concerns and the demand for sustainable processing technologies have stimulated interest in alternative green solvents like DESs.

Table 3 provides an overview of the applications of various DESs for the extraction of phytochemicals from by-products of the oil industry.

### 3.1. Phenolics

Phenolic compounds constitute one of the most important groups of plant secondary metabolites and are widely distributed in fruits, vegetables, seeds, and other plant-derived materials. Structurally, they are characterized by the presence of one or more hydroxyl groups attached to an aromatic ring and include diverse classes such as phenolic acids, flavonoids, tannins, and lignin-derived compounds [95,96]. Owing to their strong antioxidant properties, phenolic compounds have attracted considerable attention because of their anti-inflammatory, antimicrobial, cardioprotective, neuroprotective, and anticancer activities [97,98]. Consequently, they are extensively utilized in the food, pharmaceutical, cosmetic, and nutraceutical industries. Agricultural and food processing by-products represent particularly valuable and sustainable sources of phenolic compounds, creating opportunities for waste valorization and the development of high-value bioactive ingredients [99,100].

In addition to their high extraction efficiency, DESs offer important advantages related to extract quality and safety. Several studies have demonstrated that DES-based extraction results in significantly lower co-extraction of trace metals compared with conventional solvents such as water, ethanol, or aqueous acetone. This reduces the potential contamination of phenolic extracts and contributes to their safer use in food, cosmetic, and nutraceutical applications [101,102]. Furthermore, DESs, particularly acid-based formulations, have been shown to enhance the stability of phenolic compounds during storage and thermal treatment. The protective solvent environment can limit the degradation of bioactive molecules, thereby preserving their antioxidant properties and extending the shelf life of the extracts [103].

Olive fruits and olive-processing by-products are among the richest natural sources of phenolic compounds, which are responsible not only for the characteristic bitterness and astringency of olives but also for many of their health-promoting properties. The main phenolic compounds identified in olive fruits and olive by-products include phenolic acids (mainly caffeic, ferulic, and *p*-coumaric acids), phenolic alcohols such as hydroxytyrosol and tyrosol, flavonoids, and secoiridoids, with oleuropein being the predominant phenolic constituent [104]. Given their abundance in olive fruits, pomace, leaves, and mill wastewater, olive-derived phenolics have become important targets for sustainable recovery processes, including the use of DESs.

One of the first studies investigating the application of NADESs for the recovery of phenolic compounds from olive pomace demonstrated that the combination of NADESs with innovative extraction techniques significantly enhanced extraction efficiency [44]. Choline chloride mixtures with citric acid, lactic acid, maltose, or glycerol were evaluated in combination with homogenization-assisted extraction, microwave-assisted extraction, ultrasound-assisted extraction, and high hydrostatic pressure-assisted extraction. Among the tested solvents, ChCl:Citric acid and ChCl:Lactic acid systems showed the highest extraction performance, yielding extracts with elevated total phenolic content and antioxidant activity. In particular, Homogenization combined with the citric acid-based NADES extraction provided the best overall results and outperformed the other extraction techniques. Furthermore, HPLC analysis revealed that NADES-based systems were generally more effective than conventional solvents, such as aqueous ethanol and water, for the recovery of a broad spectrum of olive phenolics.

The effective combination of NADESs and microwave-assisted extraction (MAE) was presented in the work of Sonia Bonacci et al., demonstrating the recovery of phenolic compounds from olive leaves and ripe drupes coming from the production of the olive oil. Analytical results confirmed that MAE enhances the process performance, and among the tested systems, glycerol-based NADES showed the highest efficiency, even outperforming water used as the conventional solvent, while the DES composed of choline chloride and urea proved to be the least effective extraction medium. Among the identified phenolic compounds, most are derived from oleuropein [46].

The study of Alifaki and coworkers demonstrated the recovery of phenolics from olive mill wastewater. Among the investigated methods, microwave-assisted extraction and ohmic-assisted extraction showed the highest efficiency, yielding the greatest total phenolic content while reducing extraction time by up to 93.75% compared to conventional maceration [105]. Ohmic-assisted extraction is based on the passage of alternating electric current through the sample, generating heat through electrical resistance and thereby enhancing extraction efficiency. Microwave-assisted extraction was particularly noteworthy, exhibiting high antioxidant activity (0.93 mg DPPH/g extract and 9.99 mg Trolox/g extract) and a lower environmental impact, as evidenced by a substantial reduction in the chemical oxygen demand of the treated effluent. In addition, the solvent composition significantly influenced extraction performance, with the choline chloride–acetic acid DES proving to be the most effective green solvent by providing the highest total phenolic content and antioxidant activity among the tested formulations.

The study of Pietrangeli et al. demonstrated the potential of Supramolecular Deep Eutectic Solvents (SupraDESs) for the extraction of phenolics from olive pomace [77]. The optimized extraction system consisted of ammonium acetate and lactic acid supplemented with 1.8% *β*-cyclodextrins (*β*-CDs), which enhanced the extraction efficiency through the formation of inclusion complexes with phenolic compounds. Compared with conventional green solvents such as water and ethanol, the SupraDES showed superior extraction performance (expressed as mg of gallic acid equivalent per gram of dry weight of the sample) and enabled the recovery of a broader range of bioactive molecules. Specifically, the extraction yield increased by approximately 26% compared with the DES without *β*-CDs and by about 104% compared with conventional solvents such as ethanol and water. Moreover, the extraction process was successfully scaled up, demonstrating its potential for industrial implementation in accordance with the principles of green chemistry and the circular economy. However, despite its advantages, the method presents some limitations, including the difficulty of releasing phenolic compounds from the *β*-CD complexes for subsequent applications.

Mir-Cerdà et al. developed an efficient laboratory-scale purification strategy for olive leaf extracts obtained using a ChCl:Glycerol NADES [106]. The purification process employed non-functionalized polymeric resins to remove co-extracted sugars and solvent residues while preserving valuable phenolic compounds. Two sequential water-washing steps effectively eliminated residual solvent with minimal phenolic losses, whereas elution with an ethanol–water mixture (70:30, *v*/*v*) enabled efficient recovery of the target bioactive compounds. The tested resins exhibited complementary selectivity: PAD900 resin favored the recovery of larger flavonoids, while MN202 showed higher affinity for smaller polar phenolics. The purified fractions displayed high antioxidant activity and improved extract quality. Furthermore, both the resins and the NADES could be reused over multiple cycles without significant loss of performance, highlighting the sustainability and industrial potential of the proposed purification workflow.

The combination of NADES and MAE was successfully applied for the recovery of phenolic compounds from sunflower pomace. Among the tested solvents, a ChCl:Urea:Water (1:2:4) formulation exhibited the most favorable extraction performance and physicochemical properties. Under optimized MAE conditions, the obtained extracts showed higher total phenolic content and stronger antioxidant activity than those prepared using conventional solvents, like ethanol, methanol, water or acetone. Moreover, the NADES extracts were directly incorporated into strawberry–yogurt smoothie beverages, where they significantly enhanced the antioxidant potential of the beverages, increasing total antioxidant capacity by up to 68.6% and free radical scavenging activity by up to 67.9% [78].

The study by Keskin et al. demonstrated the potential of combining ultrasound-assisted extraction (UAE) with NADES for the recovery of phenolic compounds from sunflower seed shells, an underutilized agro-industrial by-product [23]. Chlorogenic acid was identified as the predominant phenolic compound in all extracts. Among the tested solvents, Glucose:Lactic acid (1:5) and Glycerol:Lactic acid (1:3) exhibited the highest extraction efficiencies, particularly for chlorogenic acid, the predominant phenolic compound identified in sunflower seed shell extracts. Most NADES extracts showed higher total phenolic content, antioxidant capacity, and bioaccessibility than conventional ethanolic extracts. Although roasting reduced the bioaccessibility and antioxidant potential of the extracts, the overall results confirmed that NADES combined with UAE is an effective green extraction strategy capable of enhancing both the recovery and gastrointestinal availability of phenolic compounds.

The study of Koh et al. demonstrated the extraction of both phenolic compounds and carotenoids from oil palm leaves [79]. Among the tested formulations, ChCl:Xylose (1:1) provided the highest total phenolic content, reaching 20.24 mg gGAE/g fresh oil palm leaves. In contrast, the highest total carotenoid content was obtained with ChCl:Xylitol (1:1), yielding 325.94 μg/g fresh oil palm leaves. The study also highlighted the role of solvent acidity, as ChCl:Fructose (1:2) with a pH of 5.82 outperformed ChCl:Glucose (1:1) with a pH of 6.72, despite their similar polarity and viscosity, indicating that lower pH enhances the solubility and stability of phenolic compounds during extraction. The stability of the prepared DES formulations during storage was also evaluated. While ChCl:Glucose (1:1), ChCl:Fructose (1:1), ChCl:Fructose (1:2), and ChCl:Glycerol (1:4) exhibited high stability and remained homogeneous throughout the storage period, the ChCl:Xyl (1:1) system underwent recrystallization after only seven days, indicating reduced stability. Furthermore, ChCl:Galactose formulations showed phase separation during storage, demonstrating inherent instability under ambient conditions.

### 3.2. Proteins

Food proteins are commonly extracted from complex biomass matrices containing lipids, fibers, minerals, and phenolic compounds. Protein extraction methods can be broadly classified into dry and wet fractionation [107]. Dry fractionation relies on differences in particle size, density, or electrical properties and preserves protein functionality due to mild processing conditions, although it generally yields protein concentrates of lower purity. In contrast, wet fractionation involves protein solubilization in a suitable solvent, followed by separation, concentration, and drying steps, enabling the production of highly purified protein isolates [108]. The efficiency of protein extraction is strongly influenced by factors such as solvent type, pH, ionic strength, temperature, and the intrinsic properties of proteins, including their size, structure, amino acid composition, and cellular localization [109,110]. Since proteins differ substantially among different plant sources, extraction strategies must be tailored to their specific physicochemical characteristics to maximize recovery while preserving functionality.

The mechanism of protein extraction by DESs differs from conventional alkaline extraction. In traditional processes, protein solubilization is primarily governed by pH-dependent changes in protein surface charge. At pH values above the isoelectric point, proteins acquire a net negative charge, which enhances electrostatic repulsion between protein molecules and increases their interaction with the aqueous medium, thereby promoting solubilization [109,111]. Although the exact mechanisms governing DES–protein interactions remain incompletely understood, current evidence suggests that hydrogen bonding is the dominant factor controlling protein dissolution in these systems. DES-mediated extraction appears to rely predominantly on the formation of extensive hydrogen-bonding networks between solvent components and protein molecules rather than on pH-induced electrostatic effects. These interactions facilitate protein solubilization while contributing to the stabilization of protein structure [108,112]. Furthermore, certain DESs are capable of partially disrupting the lignocellulosic matrix surrounding intracellular proteins, thereby improving solvent accessibility and enhancing protein release from plant tissues [108].

Grudniewska and coworkers demonstrated the potential of DES Glyceline as a green extraction medium for the recovery of proteins from rapeseed and evening primrose cakes [80]. Protein-rich precipitates were obtained by adding water as an antisolvent to the DES extracts, and their protein content was confirmed using multiple analytical techniques. The recovery of protein-rich precipitates increased with treatment temperature, reaching maximum yields of 20% for rapeseed cake and 35% for evening primrose cake at 140 °C. Furthermore, the protein content of the obtained extracts ranged from 40 to 50%, representing an enrichment of up to 20% compared with the original oilseed cakes. Moreover, the rapeseed fractions were enriched in cruciferin proteins and exhibited a light color, making them attractive for food applications.

Karabulut et al. evaluated DES-based extraction as an alternative to conventional alkaline extraction acid precipitation for recovering proteins from sunflower meal [81]. Among the tested formulations, ChCl:Glycerol and ChCl:Glucose achieved the highest soluble protein yields, reaching 12.34 and 12.88 g/100 g, respectively. Compared with alkaline extraction, DES-based methods better preserved protein structure and functionality, resulting in improved physicochemical properties. In particular, the ChCl: Glycerol isolate exhibited the highest protein solubility (36.20%) and degree of hydrolysis (57.26%), indicating enhanced digestibility, while maintaining high protein bioaccessibility (~70%). Structural analyses further revealed that DES extraction preserved secondary protein structures and reduced aggregation relative to alkaline treatment.

A comparison of protein extraction using DESs and the conventional alkali–acid extraction method was performed on defatted soybean cake. Among the investigated systems, ChCl:Glycerol DESs consistently outperformed ChCl:Polyethylene glycol formulations, owing to their higher polarity, lower viscosity, and greater capacity to solubilize proteins. The highest extraction efficiency was achieved using a ChCl:Glycerol molar ratio of 1:3, yielding 0.28 g of protein per gram of raw material with a protein content of approximately 72% [82]. Compared with the reference soy protein, the DES-extracted protein exhibited lower solubility and water hydration capacity, indicating structural modifications induced during extraction. However, it maintained a comparable foaming capacity and showed significantly improved emulsifying stability, likely due to increased surface hydrophobicity. While reduced foam stability and solubility may limit certain applications, these characteristics can be advantageous in products requiring smooth texture, rapid foam dissipation, and stable emulsions. Overall, the extracted protein demonstrated functional properties suitable for a wide range of food applications.

Similar observations regarding the technological properties of isolated proteins were reported for hazelnut meal isolates [83]. Although conventional extraction achieved higher protein recovery, it resulted in greater protein denaturation and aggregation, negatively affecting solubility and digestibility. In contrast, ultrasound-assisted DES extraction preserved native-like secondary structures, leading to improved solubility (75–76%), enhanced emulsifying properties, and higher in vitro digestibility (up to 76%). However, alkaline-extracted proteins exhibited superior foaming capacity and foam stability. These findings indicate that, as observed for soybean proteins, DES-based extraction can produce protein isolates with enhanced functional properties, particularly for applications requiring high solubility, emulsification performance, and digestibility, while alkaline extraction may remain advantageous for aerated food systems.

Seabuckthorn seed meal is a protein-rich by-product of the oil extraction industry. The results of protein isolation using DESs based on choline chloride combined with glycerol, oxalic acid, and urea were compared with those obtained using conventional extraction [84]. Although alkaline extraction achieved a higher protein recovery from seabuckthorn seed meal (56.9%) and produced isolates with a greater protein content (73.1%) than the tested DES systems (31.0–41.4% recovery and 64.3–67.5% protein content), DES-extracted proteins exhibited several advantageous nutritional and structural characteristics. In particular, DES treatment resulted in proteins with lower *β*-sheet content, higher *β*-turn content, and increased levels of total and essential amino acids. Among the investigated solvents, ChCl:Urea showed the best performance, yielding proteins with a molecular weight distribution closely resembling that of the native raw material and the highest in vitro digestibility (54.2% by pepsin), indicating superior preservation of protein quality despite the lower extraction efficiency.

Sacha inchi seed meal (SIM) is a by-product of the oil extraction industry and contains a high protein content. Sharma et al. present a novel ultrasound-assisted DES extraction method for SIM protein using a sequential process [85]. Four different DESs were evaluated, among which ChCl:Glycerol showed the most promising results and was selected for further analysis. The sequential ultrasound–ChCl:Glycerol process enabled a high crude protein recovery (77.43%) compared to ultrasound alone (29.21%) or DES alone (58.32%). The extracted SIM protein exhibited high solubility (94.39% at alkaline pH) and the highest in vitro digestibility (71.16%) using digestive enzymes (pepsin and trypsin). SDS-PAGE and FTIR analyses confirmed the preservation of protein structure and functional groups.

### 3.3. Tocopherols

Tocols (vitamin E), including tocopherols and tocotrienols, are lipophilic bioactive compounds characterized by a chromanol ring and a hydrophobic side chain. Due to their antioxidant properties and health benefits, they are valuable targets for extraction and recovery from natural sources. Deodorizer distillates (DDs), generated during vegetable oil refining, constitute an attractive and cost-effective source of tocols, as a significant proportion (1–20%) of these compounds is concentrated in the distillate fraction during the refining process [113].

The application of DESs for the recovery of tocopherols from soybean oil deodorizer distillate has been investigated as a sustainable alternative to conventional extraction methods. A series of choline chloride-based DESs containing various hydrogen bond donors (acetic acid, malonic acid, ethylene glycol, glycerol, phenol, and cresol isomers) were evaluated. Among the tested systems, the DES composed of ChCl:*p*-Cresol exhibited the highest extraction efficiency, enabling the recovery of 77.6% of total tocopherols under mild conditions (25 °C, 0.5 min) [86]. The enhanced extraction performance was attributed to π–π interactions between the aromatic structure of the phenolic DES and the chromanol ring of tocopherols. Subsequent re-extraction with *n*-hexane afforded near-quantitative recovery of α-, γ-, and δ-tocopherols (>98%), highlighting the potential of phenolic DESs as efficient and environmentally benign solvents for the valorization of vegetable oil refining by-products.

When a series of tetrabutylammonium chloride (TBAC)-based DESs, comprising polyols, amino acids, and phenolic compounds as HBDs, were investigated for the extraction of tocopherols from soybean oil deodorizer distillate methyl esters, the TBAC:4-Methylphenol DES demonstrated the highest extraction efficiency [87]. Under optimized conditions, this system achieved extraction efficiencies of 85.0%, 99.1%, and 98.0% for α-, γ-, and δ-tocopherol, respectively, corresponding to a total tocopherol recovery of 97.5%. The superior extraction efficiency of phenol-based DESs was attributed to the synergistic contribution of hydrogen-bonding and π–π interactions between the DES components and tocopherol molecules. Furthermore, the molecular structure of the phenolic HBD significantly influenced extraction selectivity toward individual tocopherol homologs, demonstrating the critical role of DES composition in the recovery of lipophilic bioactive compounds.

An intensified recovery process for vitamin E from methylated oil deodorizer distillates (MODDs) was developed based on the in situ DES formation between tocopherols and an organic salt. In MODDs, vitamin E acts as a weak hydrogen bond donor, whereas methyl linoleate behaves as a weak hydrogen bond acceptor, limiting their ability to spontaneously form DESs. Therefore, the selection of an appropriate organic salt is crucial for promoting DES formation and facilitating selective vitamin E extraction. Three chloride-based salts (choline chloride, tetraethylammonium chloride—[N_2_,_2_,_2_,_2_]Cl, and tetrabutylammonium chloride—[N_4_,_4_,_4_,_4_]Cl) were evaluated with respect to their structural characteristics and interaction strength with α-tocopherol. Due to its larger free volume and stronger associative interactions with tocopherol molecules, [N_4_,_4_,_4_,_4_]Cl exhibited the highest propensity for DES formation and was identified as the most effective extraction agent. The extraction mechanism relied on the formation of a DES between tocopherols and [N_4_,_4_,_4_,_4_]Cl, with the phase behavior governed by the salt-to-tocopherol ratio and temperature. Application of the optimized process enabled the recovery of vitamin E with a purity of 99.63% from model MODDs and >79.18% from industrial MODDs [88].

To further enhance the selectivity of vitamin E recovery from deodorizer distillates, a biphasic extraction system combining tetrabutylammonium chloride ([N_4_,_4_,_4_,_4_]Cl) with an organic solvent was developed. The study was initially conducted using a model system composed of α-tocopherol and methyl linoleate, representing vitamin E and fatty acid methyl esters (FAMEs), respectively, which are the major constituents of MODDs [114]. In this approach, the organic solvent preferentially extracted methyl linoleate, while [N_4_,_4_,_4_,_4_]Cl selectively recovered α-tocopherol through in situ deep eutectic solvent formation. Ester-, arene-, and alkane-based solvents were screened using the COSMO-RS model, considering both their interactions with methyl linoleate and the solubility of the organic salt. Hexane was identified as the most suitable solvent due to its strong affinity for methyl linoleate and limited capacity to dissolve [N_4_,_4_,_4_,_4_]Cl, which promoted efficient phase separation. The enhanced extraction selectivity was attributed to the nonpolar nature of hexane, as confirmed by σ-profile and σ-potential analyses. Experimental validation demonstrated that the hexane/[N_4_,_4_,_4_,_4_]Cl biphasic system significantly improved vitamin E selectivity compared with single-phase extraction, with the separation performance further enhanced by increasing the hexane dosage and the initial vitamin E concentration in the feed.

### 3.4. Carbohydrates

A study conducted on flaxseed cake, a by-product of flaxseed oil production, investigated the use of NADES for the extraction of uronic acid-rich polysaccharides, particularly pectins. Among the tested solvent systems, a ChCl:Citric acid NADES ratio provided the highest pectin recovery (36.88 mg/g), the greatest uronic acid content, and the lowest level of protein impurities, indicating superior extract purity. Structural characterization revealed that the recovered polysaccharides consisted predominantly of homogalacturonan with xylogalacturonan domains, while physicochemical analyses confirmed their anionic nature and pH-dependent behavior typical of pectic biopolymers [89]. The results demonstrated that NADES-based extraction outperformed conventional extraction approaches in terms of selectivity and product quality, highlighting the potential of these green solvents for the sustainable recovery of high-value polysaccharides from flaxseed processing residues. Furthermore, the extracted pectin exhibited properties suitable for applications in food formulations, biodegradable packaging materials, and pharmaceutical delivery systems, supporting the valorization of flaxseed cake within a circular bioeconomy framework.

Another example of the utilization of flaxseed by-products for the production of bioactive oligosaccharides was reported by Hu et al., who developed a sustainable extraction strategy combining hydrophobic deep eutectic solvent-based three-phase partitioning with enzymatic hydrolysis [90]. A Menthol:Octanoic acid hydrophobic DES enabled the efficient recovery of water-soluble polysaccharides while simultaneously removing protein impurities, thereby simplifying downstream purification. Subsequent enzymatic hydrolysis with pectinase produced flaxseed gum oligosaccharides (FGOS) with a low molecular weight (1.75 kDa). Biological evaluation demonstrated that FGOS exhibited significant anti-inflammatory activity in LPS-stimulated RAW 264.7 macrophages by reducing nitric oxide production and suppressing the expression of pro-inflammatory mediators, including TNF-*α*, IL-1*β*, IL-6, and iNOS (an inflammation-associated gene that is strongly upregulated in response to lipopolysaccharide stimulation and is closely linked to the excessive production of NO).

The potential of DES-assisted valorization has also been demonstrated for hemp seed cake flour (HSCF), a by-product generated during hemp oil production. In a recent study, HSCF was pretreated with a ChCl:Glycerol DES, followed by enzymatic hydrolysis, to improve its nutritional and technological properties. The optimal pretreatment conditions (120 °C and a biomass-to-DES ratio of 1:8) resulted in approximately 32% lignin removal while maintaining biomass recovery above 85%. Subsequent enzymatic hydrolysis further reduced the insoluble fiber fraction and promoted the release of xylooligosaccharides with potential prebiotic properties [91].

Another example of DES-assisted valorization of oilseed processing residues was presented by researchers investigating the extraction of polysaccharides from oilseed pumpkin flesh [92]. Using a deep eutectic solvent-based extraction approach, crude polysaccharides were recovered with a yield of up to 10.9%, followed by purification to obtain a homogeneous fraction (PPOP-3). Structural analyses revealed that PPOP-3 was a highly branched acidic polysaccharide composed of nine monosaccharides and exhibited a molecular weight of 2.02 × 10^5^ Da. In vitro assays demonstrated notable antioxidant activity, highlighting its potential as a bioactive ingredient. Importantly, the study adopted an integrated biorefinery concept in which the solid residues remaining after polysaccharide extraction were converted into magnetic biochar. The resulting material exhibited a porous structure and effectively adsorbed carotenoids during pumpkin seed oil refining, while maintaining good reusability. These findings demonstrate that DES-based extraction can support the simultaneous recovery of high-value polysaccharides and the production of functional adsorbent materials, contributing to the comprehensive and sustainable valorization of oilseed processing by-products.

Several studies have demonstrated the potential of DES-assisted extraction for the recovery of bioactive polysaccharides from *Camellia oleifera* by-products. Feng et al. investigated the extraction of polysaccharides from *C. oleifera* fruit shells, an abundant residue of the tea oil industry [93]. Among 43 tested DES formulations, choline chloride-based systems containing propionic acid and 1,3-butanediol achieved the highest extraction efficiency, yielding up to 150.27 mg/g of polysaccharides, which was approximately 1.5 times higher than that obtained by conventional hot-water extraction. The extracted polysaccharides consisted of rhamnose, arabinose, galactose, glucose, xylose, mannose, galacturonic acid, and glucuronic acid, with molecular weights ranging from 8.9 × 10^4^ to 74.3 × 10^4^ Da. Moreover, DES-derived polysaccharides exhibited enhanced antioxidant and hypoglycemic activities compared with water-extracted counterparts.

Similarly, Qilu Xue et al. explored the extraction of polysaccharides from *C. oleifera* leaves, a by-product generated during routine pruning [94]. Among eleven tested betaine-based DESs, a Betaine:Lactic acid system proved the most effective, achieving a polysaccharide recovery of 19.01%, which was approximately 2.5-fold higher than that obtained using conventional hot-water extraction. Although the monosaccharide composition of the extracted polysaccharides was comparable to that of water-extracted fractions, DES extraction resulted in significantly lower molecular weight and smaller particle size. These structural modifications were associated with improved thermal stability, emulsifying properties, and antioxidant activity, with DPPH radical scavenging exceeding 90%. Collectively, these studies demonstrate that DESs not only improve polysaccharide recovery from *C. oleifera* processing residues but can also modulate their structural characteristics and enhance their biological and functional properties, thereby expanding their potential applications.

## 4. Deep Eutectic Solvents in Oil Purification

DESs have recently attracted considerable attention not only as extraction media for bioactive compounds but also as sustainable agents for oil purification and the treatment of technological waste generated during vegetable oil processing. Conventional refining processes often require large amounts of organic solvents, water, and energy, leading to the generation of secondary pollutants and significant environmental burdens. In this context, DESs represent a promising green alternative.

One of the most extensively investigated applications of DESs in oil refining is the removal of undesirable compounds such as free fatty acids, pigments, phospholipids, sulfur-containing compounds, persistent organic pollutants, parabens, mycotoxins, and trace metals. The strong hydrogen-bonding interactions and adjustable polarity of DESs enable selective separation of impurities while minimizing the loss of valuable lipid components. DES-based purification methods have been studied for various vegetable oils, including soybean, rapeseed, sunflower, and palm oils.

Conventional palm oil refining relies on steam stripping to reduce free fatty acid content, but this process can lead to the loss of valuable nutraceutical compounds such as tocopherols and carotenoids. As a greener alternative, NADESs based on betaine as the HBA and various polyalcohols as the HBD were investigated for the extraction of palmitic acid, the predominant free fatty acid in palm oil [115]. Among the tested systems, the Betaine:1,2-Butanediol NADES exhibited the highest extraction efficiency, achieving a palmitic acid extraction yield of 60% (*w*/*w*) with a distribution coefficient of 0.75. The results demonstrated that lower-viscosity NADESs generally provided higher extraction yields when the polarity of those solvents remained similar.

DESs and ionic liquids have also been investigated as potential green extractants for the removal of persistent organic pollutants (POPs), including polychlorinated dibenzo-*p*-dioxins, dibenzofurans, and polychlorinated biphenyls, from edible oils and fats. Using the COSMO-SAC predictive modeling approach, the authors screened 217 DES formulations composed of 24 hydrogen bond acceptors and 62 hydrogen bond donors to identify systems with high affinity toward these contaminants [116]. Although no single DES was predicted to efficiently extract all 27 target POPs, several formulations demonstrated promising performance. In particular, ChCl:Sucrose and Benzyl tripropylammonium chloride:Phenol DESs were predicted to remove 25 of the 27 investigated pollutants. Overall, eight choline chloride-based DESs, six benzyl-based DESs, and one phosphonium-based DES showed high extraction potential for most POPs. The modeling results also indicated that the solubility of POPs in DESs increased by more than 1.3-fold compared with conventional systems, highlighting their suitability as extraction media. Importantly, several of the most promising DESs contained polysaccharide-based hydrogen bond donors, which may reduce toxicity concerns and enhance their applicability for the purification of edible oils and fats.

An interesting development in the field of green extraction is the use of water-based deep eutectic solvents (WDESs). In one study, a WDES composed of tetrabutylammonium chloride and water at a molar ratio of 1:5 was developed and applied to ultrasound-assisted liquid–liquid microextraction of parabens (methylparaben, ethylparaben, propylparaben and butylparaben) from edible oils prior to HPLC analysis [117]. Water acted not only as a hydrogen bond donor but also as a component that tailored the physicochemical properties of the solvent system and facilitated the in situ formation of DES interactions with the target analytes. The optimized TBAC–water system exhibited significantly higher extraction efficiency than water alone and even outperformed a conventional DES based on choline chloride and urea. The developed method showed excellent analytical performance, including low detection limits (0.2–0.4 μg/L), good precision (RSD ≤ 5.8%), and high recoveries (85.1–106.8%). The authors highlighted that the simplicity, low cost, high extraction efficiency, and environmental friendliness of water-based DESs make them promising alternatives for the extraction of parabens and potentially other hydrophobic contaminants from edible oil matrices.

Another innovative approach involves the use of supramolecular DESs for the extraction and determination of trace elements in edible vegetable oils like olive, soybean, canola, corn, camellia, peanut or sunflower oil. A SupraDES based on 2-hydroxypropyl β-cyclodextrin and lactic acid was successfully applied in vortex-assisted dispersive liquid–liquid microextraction for the preconcentration of Cu, Zn, and Mn, followed by inductively coupled plasma optical emission spectrometry (ICP-OES) analysis [118]. Under optimized conditions, extraction efficiencies exceeded 99% for all three elements, with enrichment factors ranging from 21 to 35 and detection limits ranging from 0.89 to 1.30 μg/L. The authors emphasized that SupraDESs combine the advantages of conventional DESs with the unique host–guest properties of cyclodextrins, resulting in excellent extraction performance.

Another approach for the determination of trace elements in edible oils combined deep eutectic solvents with vortex-assisted dispersive liquid–liquid microextraction (DLLME), enabling efficient preconcentration of Fe, Cu, and Pb prior to analysis by microwave-induced plasma optical emission spectrometry (MIP-OES) [119]. The method employed a ChCl:Ethylene glycol DES as the extraction medium and demonstrated excellent analytical performance, including low detection limits (0.7–3 μg kg^−1^), good repeatability (CV < 9.2%), and recoveries close to 100%, indicating negligible matrix effects. The authors emphasized that the use of small volumes of DES significantly reduced reagent consumption and waste generation, while vortex-assisted dispersion eliminated the need for additional disperser solvents, thereby enhancing both the environmental sustainability and efficiency of the procedure. Furthermore, coupling DES-based DLLME with MIP-OES provided a cost-effective alternative to more expensive elemental analysis techniques commonly used in the food industry. According to the AGREEprep assessment [120], the method demonstrated satisfactory green analytical performance and represents a rapid, simple, economical, and environmentally friendly strategy for monitoring trace metals in edible oil samples.

A NADES-based liquid–liquid microextraction method coupled with HPLC-FLD/DAD was developed for the simultaneous extraction and quantification of four mycotoxins: deoxynivalenol, alternariol, ochratoxin A, and zearalenone [121]. The optimized extraction system, based on ChCl:Urea containing 16.4% water, enabled efficient co-extraction of the target compounds from various edible oils without the use of hazardous organic solvents or additional clean-up steps. Recoveries ranged from 100% to 103% for most analytes (except for zearalenone), with relative standard deviations below 9%, meeting European Union validation requirements. The method was successfully applied to several vegetable oils, including sunflower, olive, corn, peanut, and rice bran oils. Importantly, the authors emphasized that the analytical performance of the proposed NADES-based method compared favorably with that of more sophisticated instrumental approaches, such as UPLC–MS/MS.

Hydrophobic natural deep eutectic solvents have also been explored for the valorization of olive oil mill wastewaters (OOMWs), one of the major by-products of olive oil production. Although OOMWs are rich in valuable polyphenols with potential nutraceutical and cosmeceutical applications, their utilization is limited by the presence of phenol, a toxic compound that requires removal before further processing. To address this challenge, hydrophobic NADESs composed of combinations of octanoic acid, dodecanoic acid, and menthol were evaluated as extraction media for the selective removal of phenol from a model OOMW solution [122]. For the Octanoic:Dodecanoic acid NADES, the octanoic acid acted as the hydrogen bond donor and dodecanoic acid as the hydrogen bond acceptor. In the menthol-based NADESs, menthol served as the HBA, while octanoic or dodecanoic acid functioned as the HBD. Among the tested systems, the Octanoic:Dodecanoic acid NADES exhibited the best extraction performance, effectively separating phenol while preserving tyrosol, used as a representative beneficial polyphenol. The extraction process was most efficient at neutral pH, highlighting the importance of operating conditions on the selectivity of the process.

## 5. Practical Aspects

Despite the numerous advantages offered by DESs for the extraction of bioactive compounds from by-products of the oil processing industry, several important challenges must still be addressed before these systems can be widely implemented on an industrial scale. Among the most significant barriers are the regulatory uncertainties surrounding their use, as comprehensive legal frameworks governing DES-based processes and products are still lacking [123,124]. Although many DES components are recognized as safe individually, their classification, approval pathways, residue limits, and labeling requirements as eutectic mixtures remain insufficiently defined [125]. Addressing these regulatory issues will be essential to facilitate the broader adoption of DES technologies in food, nutraceutical, and related industrial applications.

To address the sustainability issue, a novel assessment framework termed the *DES Ecoscale* was recently proposed [126]. This quantitative tool evaluates DES sustainability through five key dimensions, including component sustainability, synthesis conditions, physicochemical properties, application-related aspects, and end-of-life behavior. Validation of the framework demonstrated significant differences between individual DESs, with NADESs composed of GRAS-approved components achieving substantially higher sustainability scores than many synthetic DESs. Importantly, the authors showed that conventional green chemistry metrics may fail to identify sustainability concerns associated with the chemical nature of DES constituents, whereas the *DES Ecoscale* enables a more comprehensive and transparent evaluation. Such approaches may play an important role in supporting the implementation of Safe and Sustainable by Design (SSbD) principles and in guiding the future development of DESs for industrial applications [124].

While the *DES Ecoscale* provides a useful framework for assessing the intrinsic sustainability of DESs, the overall environmental and economic performance of DES-based extraction processes is also strongly influenced by process integration, solvent recovery, and downstream processing. These aspects ultimately determine the commercial feasibility of DES-based technologies, yet, to the best of our knowledge, no published techno-economic analyses (TEAs) or life cycle assessments (LCAs) have specifically evaluated DES-based oilseed biorefineries.

Nevertheless, valuable insights into their economic and environmental performance can be drawn from studies on DES-assisted biorefineries utilizing other agricultural feedstocks. For example, techno-economic analysis performed by Kumar et al. demonstrated that targeting multiple high-value, low-volume products, rather than relying on high-volume, low-value commodities, substantially improves the economic viability of DES-based biorefineries [127]. Furthermore, efficient solvent recycling and reuse reduced raw material operating costs by up to 65%, highlighting solvent recovery as a critical requirement for economically feasible process design.

In line with this, it has been demonstrated that the overall environmental performance also strongly depends on the downstream processing strategy, particularly the solvent recovery and product purification steps. For example, a recent combined TEA and LCA study on cellulose nanocrystal production from agricultural biomass demonstrated that implementing efficient solvent recovery reduced unit production costs by approximately 25% while decreasing the Global Warming Potential (GWP) by more than 70%, together with substantial reductions in toxicity-related impact categories [128]. Sensitivity analyses further confirmed the economic robustness of the process under varying market conditions, emphasizing solvent recyclability as a key factor for both economic viability and environmental sustainability. In contrast, a recent LCA by Bouhzam et al. on polyphenol extraction from spent coffee grounds showed that, despite achieving higher extraction yields, the DES-based process exhibited greater environmental impacts than extraction with 20% ethanol, even when assuming a 90% DES recovery rate [129]. Further on, a combined economic and environmental assessment of DES-assisted extraction of plant bioactives reported that ultrasound-assisted extraction of anthocyanins from grape pomace using DESs resulted in higher capital and operating costs, as well as slightly greater environmental impacts, than the corresponding ethanol-based process [130]. The higher environmental burden was primarily attributed to the complex downstream purification, particularly the large water demand during adsorption chromatography. Importantly, when the process was redesigned to produce ready-to-use DES extracts without the purification of the target compounds, the sustainability and economic indicators improved substantially, demonstrating that process configuration and the intended product format can have a decisive influence on the overall sustainability of DES-based processes.

In summary, the available evidence indicates that the sustainability and economic competitiveness of DES-based biorefineries are determined not only by the intrinsic properties of the solvents but also by process integration, efficient solvent recovery, and the design of downstream operations. These aspects should therefore be considered central elements in the development and scale-up of DES-based oilseed biorefinery processes.

Among these factors, efficient solvent recovery and recycling remain one of the major technological challenges for the large-scale implementation of DES-based extraction processes [68]. Various recovery techniques have been investigated, including anti-solvent precipitation, crystallization, membrane filtration, solid–liquid extraction, liquid–liquid extraction, short-path distillation, supercritical fluid extraction, and density-based separation [80,131,132,133,134,135,136]. Some of the listed recovery approaches have been reported to achieve DES recovery rates exceeding 90%. Anti-solvent precipitation is particularly attractive because of its high recovery efficiency and ease of scale-up; however, it requires substantial amounts of auxiliary solvents and additional energy for solvent regeneration. Liquid–liquid extraction is relatively simple and energy-efficient but is limited by the partitioning behavior of the target compounds and the use of organic solvents, whereas solid–liquid extraction enables selective separation without toxic solvents but is constrained by the high cost of adsorbents and limited industrial scalability [68]. Furthermore, the accumulation of impurities and dissolved biomass components during repeated recycling cycles often leads to reduced extraction efficiency and product yields. Consequently, the development of cost-effective, energy-efficient, and scalable DES recovery technologies remains a key prerequisite for their successful industrial application.

Although DES implementation presents several limitations, these drawbacks should be considered in the broader context of green extraction technologies, as no sustainable solvent system is entirely free of operational constraints. Supercritical fluid extraction (SFE), particularly using supercritical CO_2_ (SC-CO_2_), is among the most mature green extraction techniques and has been extensively investigated for the recovery of lipophilic bioactive compounds from oil-processing by-products [137,138,139]. The use of CO_2_ as a non-toxic, non-flammable, and generally recognized as safe (GRAS) solvent enables the production of solvent-free extracts while preserving thermolabile constituents due to its relatively low critical temperature (31.2 °C). Moreover, the tunable density of supercritical CO_2_ allows selective extraction of non-polar compounds such as tocopherols, phytosterols, and volatile constituents. Nevertheless, the widespread industrial implementation of SFE remains limited by the requirement for high operating pressures, substantial capital investment, and its inherently lower extraction efficiency toward polar phytochemicals, including most phenolic compounds and proteins abundant in oilseed cakes and pomaces [140]. Another environmentally friendly alternative is subcritical water extraction (SWE), which employs water under elevated temperature and pressure, thereby eliminating the need for organic solvents. SWE is characterized by enhanced mass transfer, relatively short extraction times, and the ability to recover compounds across a broader polarity range through modulation of water dielectric properties. However, the severe extraction conditions may promote hydrolysis, Maillard and caramelization reactions, or degradation of heat-sensitive bioactive compounds, while simultaneously increasing equipment costs due to the demanding operating conditions. Therefore, although DES-based extraction still faces technological challenges, its ability to efficiently solubilize polar and moderately polar metabolites under relatively mild conditions, combined with the tunability of solvent composition, makes it a highly promising complementary or alternative approach for the valorization of oilseed by-products within sustainable biorefinery concepts [140,141,142].

Because of the challenges associated with DES recovery and downstream purification, increasing attention has been directed toward process designs that eliminate the need for solvent recovery altogether. In these approaches, DESs are selected not only as extraction media but also as functional formulation components, allowing the resulting DES-based extracts to be applied directly as the final product without additional purification steps [143,144,145] or as an intermediate formulation [146,147]. Such approaches are particularly attractive when the DES components are biocompatible and safe for human use. In these cases, the extract together with the DES may serve as a ready-to-use formulation, eliminating the need for costly purification and solvent regeneration steps. This strategy has attracted considerable interest in the cosmetic, nutraceutical, and pharmaceutical sectors, where DES-based extracts can be directly incorporated into creams, gels, serums, or other functional products [130,148,149,150]. Consequently, the ready-to-use concept is increasingly regarded as a promising route to improve the economic feasibility and sustainability of DES-based extraction technologies [130]. This approach is particularly attractive because DESs often enhance the stability of extracted bioactive compounds by protecting them against oxidation, degradation, and other environmental stresses [151,152,153]. As a result, DES-based formulations may not only preserve the biological activity of the extracted compounds but also extend the shelf life of both the extracts and the final products in which they are incorporated. Considering the growing interest in ready-to-use and NADES-based extracts, the sensory evaluation of products formulated with such extracts is also of critical importance. However, relatively few studies have addressed this crucial aspect of consumer acceptance [154,155,156]. To date, most research has focused on extraction efficiency, stability, and bioactivity, while the potential effects of residual solvents and solvent-containing extracts on the taste, aroma, texture, and overall sensory quality of fortified products remain largely unexplored. Interestingly, Panić et al. presented a study in which NADES extracts rich in polyphenols were obtained from cocoa by-products and subsequently used for the fortification of chocolate milk [157]. Importantly, the sensory acceptability of the fortified beverages was evaluated using an electronic tongue combined with SIMCA multivariate analysis, representing one of the first attempts to systematically assess the sensory impact of NADES-based extracts in food products. The results demonstrated that carefully selected NADES formulations enabled efficient polyphenol extraction and could be incorporated directly into the final product without solvent removal, while maintaining satisfactory sensory acceptance. Nevertheless, such studies remain scarce, highlighting the need for more comprehensive investigations into consumer perception and sensory quality of foods enriched with DES-derived extracts before their broader industrial adoption.

## 6. Conclusions

**Figure 2:**
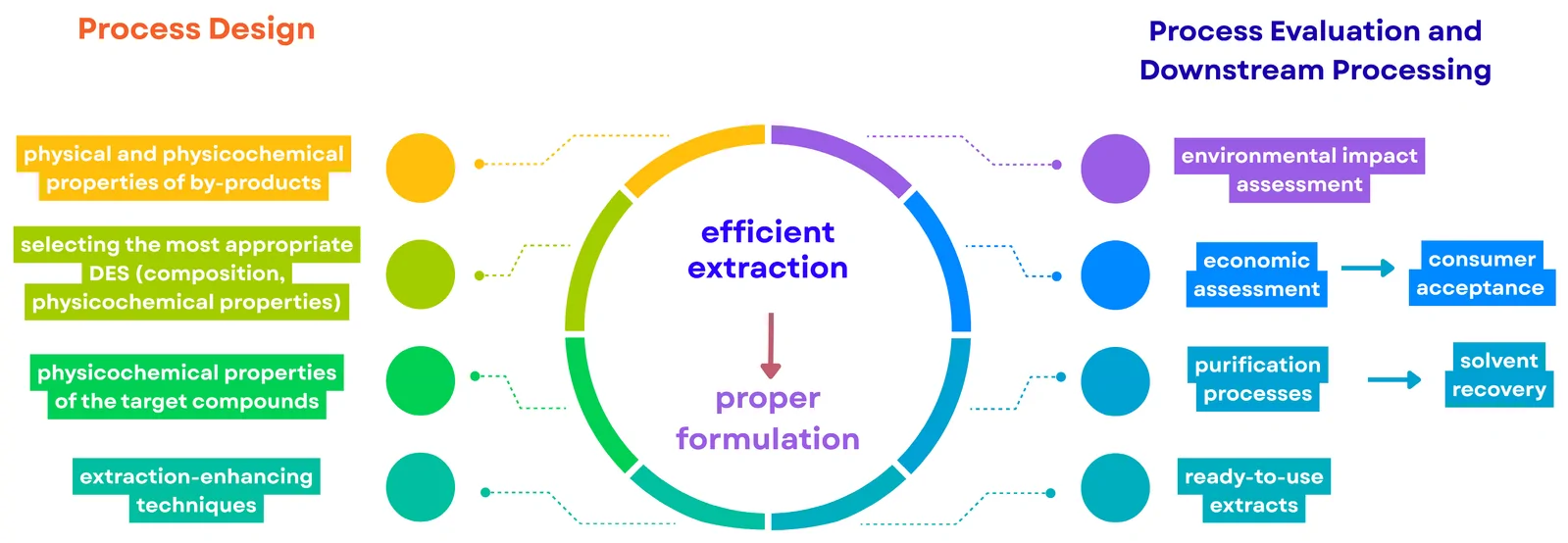
Key considerations for designing DES-based extraction processes for oilseed industry by-products.

The continuous discovery of new DESs has significantly expanded the possibilities for tailoring solvent properties to specific extraction and refining applications. By carefully selecting hydrogen bond donors and acceptors, researchers have developed solvents with diverse physicochemical characteristics, enabling the efficient recovery of a wide range of bioactive compounds and other valuable constituents from oilseed processing by-products. The key factors that should be considered when designing DES-based extraction processes for oilseed industry by-products are summarized in Figure 2.

The studies reviewed in this work demonstrate that DESs can serve not only as sustainable alternatives to conventional organic solvents but also as functional media capable of enhancing extraction selectivity, preserving bioactive compounds, and improving the nutritional and technological properties of the recovered ingredients. In some cases, DES-based extracts can be directly incorporated into food, nutraceutical, or cosmetic formulations, reducing the need for extensive downstream processing and supporting circular bioeconomy approaches.

Nevertheless, several challenges remain to be addressed, including solvent recovery and recycling, regulatory approval, toxicological validation, and the assessment of sensory acceptance of DES-containing products. Process scale-up also represents a critical challenge, as many of the extraction methods reported to date have only been demonstrated at laboratory or microscale levels. Consequently, further studies are required to evaluate mass transfer phenomena, process economics, and operational feasibility under pilot- and industrial-scale conditions. Future research should therefore focus on the rational design of DESs according to Safe and Sustainable by Design principles, the development of efficient recovery strategies, and the validation of DES-based technologies under industrial conditions. Overall, DESs represent a versatile and promising platform for transforming oilseed waste into high-value products, contributing to more sustainable and resource-efficient biorefinery systems.

## Figures and Tables

**Table 1:** Composition of selected oilseed industry by-products (g/100 g).

	Ash	Moisture	Crude Protein	Fat	Carbohydrates	Total Phenolics mg GAE/g Sample	References
Hemp	5.34	7.56	28.61	8.61	50.23	2.72	[15]
Pumpkin	9.14	4.99	59.12	12.36	14.39	1.45	[15]
Rapeseed	5.73	8.08	29.84	12.52	43.84	8.47	[15]
Sunflower	7.49	9.23	37.10	0.69	23.97	NI	[16]
Soybean meal	7.08	NI	47.42	2.83	NI	2.98	[17]
Olive cake	6.51	4.65	8.44	9.11	71.5	49.8	[18]
Olive pomace	4.48	0.93	8.75	15.61	33.28	22.73	[19]

NI—not indicated, GAE—gallic acid equivalent.

**Table 2:** Physical properties of selected DESs.

HBA	HBD	Ratio HBA: HBD	T_f_ or T_g_ [K]	Density [g/cm^3^]	Viscosity [mPa·s]	References
Choline Chloride 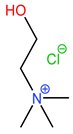	Glycerol 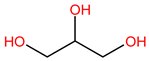	1:2	233.15	1.181 at 293.15 K	376 at 293.15 K	[32,33]
	Ethylene glycol 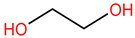	1:2	207.15	1.12 at 293.15 K	36 at 293.15 K	[32,33]
	Urea 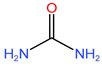	1:2	285.15	1.24 at 313.15 K	169 at 313.15 K	[34,35]
	Oxalic acid 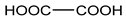	1:1	251.42	1.248 at 313.15 K	2142 at 313.15 K	[36]
	Citric acid 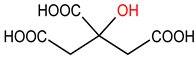	1:1	342.15	1.270 NI	9761.73 at 358.15 K	[37,38]
	Glucose 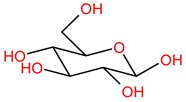	2:1	288.15	1.2115 at 358.15 K	72 at 358.15 K	[39]

NI—not indicated, T_f_—Freezing point, T_g_—Glass transition temperature.

**Table 3:** Overview of DES application in extraction of desired components from oil industry by-products.

Oilseed By-Product	DES Used (Molar Ratio)	Extracted Component/ Fraction	Other Supporting Techniques	Yield/Efficiency	References
Olive pomace	ChCl:Citric acid (1:2) ChCl:Lactic acid (1:2)	phenolic compounds, mainly oleuropein	homogenate-assisted extraction	TPC 34.08 mg GAE/g DW 28.83 mg GAE/g DW Folin–Ciocalteu assay	[44]
Olive Leaves and ripened olive drupes	ChCl:Glycerol (1:1)	phenolic compounds, mainly oleuropein	microwave-assisted extraction	Oleuropein content 88,320.90 ± 38.03 [ppm] LC-ESI-QTOF/MS analysis	[46]
Olive pomace	Ammonium acetate:Lactic acid (1:7) 1.8% of β-CDs	phenolic compounds	NI	TPC 56.98 ± 0.37 mg GAE/g DW Folin–Ciocalteu assay	[77]
Sunflower pomace	ChCl:Urea:H_2_O (1:2:4)	phenolic compounds (3-*O*-caffeoylquinic acid, 1,3-di-*O*-caffeoylquinic acid, 1,5-di-*O*-caffeoylquinic acid)	microwave-assisted extraction	TPC 0.55 mmol trolox/g DW Folin–Ciocalteu assay	[78]
Oil palm leaves	ChCl:Xylose (1:1) ChCl:Xylitol (1:1)	phenolic compounds carotenoids	homogenizer-assisted heating and stirring extraction	TPC 20.238 mg GAE/g fresh oil palm leaves, Folin–Ciocalteu assay TCC 325.94 μg/g fresh oil palm leaves, spectrophotometric method	[79]
Rapeseed cake	ChCl: Glycerol (1:2)	proteins cruciferin as a main component	NI	yield of precipitate 20%	[80]
Evening primrose cake	ChCl: Glycerol (1:2)	proteins with variable molecular weight (10–40 kDa)	NI	yield of precipitate 35%	[80]
Sunflower meal	ChCl:Glucose (1:1) ChCl:Glycerol (1:2)	proteins	ultrasound-assisted extraction	soluble protein content 12.88 and 12.34 g/100 g, respectively	[81]
Deoiled soybean cake	ChCl:Glycerol (1:3)	proteins	NI	maximum yield 0.28 g/g, 72% protein content, Kjeldahl analysis	[82]
Hazelnut meal	ChCl:Glycerol (1:2) ChCl:Glucose (2:1)	major storage proteins, including 7S and 11S globulins and 2S albumins	ultrasound-assisted extraction	yields ~200–220 mg/g efficiencies 50–55% Bradford assay	[83]
Seabuckthorn seed meal	ChCl:Urea (molar ratio NI)	proteins with variable molecular weight (11–75 kDa)	NI	protein recovery 32,3% protein content 67.5%, Dumas combustion method using a TruMac N system	[84]
Sacha inchi seed meal	ChCl:Glycerol (1:2)	proteins with variable molecular weight (12–40 kDa)	ultrasound assisted extraction	protein recovery 77.43% Kjeldahl analysis	[85]
Soybean oil deodorizer distillate	ChCl:*p*-Cresol (1:2)	α-, γ- and δ-tocopherols	vortex- assisted extraction	total tocopherol recovery 77.6%, HPLC-UV analysis	[86]
Soybean oil deodorizer distillate methyl esters	TBAC:4-methylphenol (1:1)	α-, γ-, and δ-tocopherols	vortex- assisted extraction	total tocopherol recovery 97.5%, HPLC-UV analysis	[87]
Methylated oil deodorizer distillates	[N_4,4,4,4_]Cl:α-Tocopherol (20:1)	vitamin E, α-tocopherol	NI	extraction ratio > 91.05% for all vitamin E isomers, HPLC-UV analysis	[88]
Flaxseed cake	ChCl/Citric acid (1:1)	uronic acid-rich pectins MW range of 14–500 × 10^3^ g/mol	NI	pectin recovery 36.88 mg/g, phenol-sulfuric acid method for total sugar content	[89]
Flaxseed cake meal	L-Menthol:Octanoic acid (1:1) with 20% (*w*/*v*) (NH_4_)_2_SO_4_	flaxseed gum oligosaccharides MW 1.75 kDa and a narrow dispersity	enzymatic hydrolysis	yield 83.45 mg/g, phenol-sulfuric acid method for total sugar content, monosaccharide compositions—HPLC	[90]
Hemp seed cake flour	ChCl:Glycerol (1:2)	xylooligosaccharides (xylobiose, xylotriose, xylotetraose, xylopentaose and xylohexaose)	enzymatic hydrolysis	biomass recovery > 85%, sugars quantified by HPLC with refractive index detector	[91]
Oilseed pumpkin flesh	ChCl: 1,4-Butanediol (1:4) 30 wt% H_2_O	polysaccharide fraction MW 2.02 × 10^5^ Da	NI	polysaccharide content: 10.90 ± 0.125%	[92]
*C. oleifera* fruit shells	ChCl: Propionic acid:1,3-Butanediol (2:1:1) 33.33% H_2_O	polysaccharides MW from 8.9 to 74.26 × 10^4^ Da	NI	polysaccharides yield 150.27 mg/g, phenol-sulfuric acid method for total sugar content	[93]
*C. oleifera* leaves	Betaine:Lactic acid (1:3)	polysaccharides MW 96.56 kDa	NI	polysaccharide yield 19.01%, phenol-sulfuric acid method for total sugar content	[94]

MW—molecular weight, TPC—total phenolic content, TCC—total carotenoid content, GAE—gallic acid equivalent, DW—dry weight, *β*-CDs—*β*-cyclodextrins.

## Data Availability

No new data were created or analyzed in this study. Data sharing is not applicable to this article.
